# Feasibility of longitudinal sleep monitoring among Kenyan healthcare workers using wearable technology

**DOI:** 10.21203/rs.3.rs-10460332/v1

**Published:** 2026-08-06

**Authors:** Willie Njoroge, David Andai, Minki P. Lee, Andrew Aballa, Linda Khakali, Rachel Maina, Elena Frank, Lukoye Atwoli, Zhenke Wu, Anthony K. Ngugi, Srijan Sen, Amos Bunde, Jasmit Shah, Dorcas G. Mwigereri, James Orwa, Akbar K. Waljee, Amina Abubakar, Zul Merali, Daniel B. Forger

**Affiliations:** 1 Brain and Mind Institute, Aga Khan University, Nairobi, Kenya; 2 Michigan Neuroscience Institute, University of Michigan, Michigan, United States of America; 3 Department of Medicine, Medical College East Africa, Aga Khan University, Nairobi, Kenya; 4 Department of Biostatistics, University of Michigan, Ann Arbor, Michigan, United States of America; 5 Department of Population Health, Aga Khan University, Nairobi, Kenya; 6 Center for Global Health Equity, University of Michigan, Ann Arbor, Michigan, United States of America; 7 Computing and Data Innovation Office, Aga Khan University, Nairobi, Kenya; 8 Department of Learning Health Sciences, University of Michigan Medical School, Ann Arbor, Michigan, United States of America; 9 Institute for Human Development, Aga Khan University, Nairobi, Kenya; 10 Neurosciences Unit, Kenya Medical Research Institute-Wellcome Trust Research Programme, Kilifi, Kenya; 11 Department of Mathematics, University of Michigan, Ann Arbor, Michigan, United States of America; 12 Department of Computational Medicine and Bioinformatics, University of Michigan, Ann Arbor, Michigan, United States of America

## Abstract

Although adequate high-quality sleep is essential for optimal cognitive functioning, information on sleep and circadian rhythms among healthcare workers in resource-constrained settings remains scarce. We conducted a 12-month longitudinal study across five healthcare facilities using Fitbit Inspire 2^™^ devices. Sleep duration and irregularity were broadly comparable to higher-resource populations; observed differences in circadian metrics were largely explained by age. These findings support the generalizability of wearable-based sleep and circadian algorithms across contexts when key demographic and societal factors are considered.

Sleep is essential across the lifespan^1,2^ for cognitive functioning, emotional regulation, cardiometabolic health, and overall wellbeing^3^. The National Sleep Foundation recommends that the average adult obtain 7 to 9 hours of sleep per night on a regular basis to promote optimal health^4^. Among healthcare workers (HCWs), inadequate sleep has implications not only for individual health but also for patient safety, clinical decision-making, and health system performance. Prolonged sleep deprivation and circadian disruption in HCWs have been linked to increased risk of depression, burnout, medical errors, and decreased work productivity^5,6^. Although these associations are well established in high-income countries, longitudinal and objective sleep data from HCW in low- and middle-income countries (LMICs) remain scarce.

Healthcare systems in LMICs including Kenya, face workforce shortages, and high patient volumes^7^. These structural constraints often result in long work hours, unpredictable schedules, and insufficient recovery time, increasing the risk of circadian misalignment and chronic sleep deprivation. Yet most available studies in these contexts rely on cross-sectional surveys and retrospective self-reports, which provide limited insight into day-to-day variability and are vulnerable to recall bias. Advances in digital health technologies offer new opportunities to address these gaps in healthcare delivery^8^. For example, wearable devices enable continuous, non-invasive monitoring of sleep duration, timing, and variability in real-world settings over extended periods. However, the feasibility and reliability of implementing long-term wearable monitoring in HCWs in an LMIC environment remains underexplored. Studies are needed to understand the potential barriers that may affect adherence and data quality such as limited connectivity, charging constraints, competing clinical demands, and contextual work patterns^9^.

Safeguarding the wellbeing of HCWs is critical to maintaining health system resilience, especially in LMICs confronting increasing disease burdens and patient volumes. Demonstrating the feasibility of sustained digital sleep monitoring is therefore a necessary precursor to developing scalable sleep health management and workforce scheduling interventions. As part of the Utilizing Health Information for Meaningful Impact in East Africa for Data Science (UZIMA-DS) research hub in Nairobi, Kenya, the objective of this study was to assess the feasibility of obtaining reliable sleep metrics among HCWs.

A total of 527 healthcare workers were recruited into the study. Age information was missing for seven participants; however, this did not affect their eligibility for inclusion in the sleep analyses. Of the recruited participants, 434 had Fitbit-derived sleep data available. To ensure data quality, sleep episodes lasting less than three hours were excluded, as these were unlikely to represent valid nocturnal sleep and could reflect device non-wear, incomplete recordings or naps. Following data cleaning and the application of study inclusion criteria, including the availability of complete sleep and outcome data, 228 participants (Fig.1) remained in the final analytical sample, comprising 137 females and 91 males. Among those with available data, the median age was 35.0 years (mean = 36.02 years; range = 19–62 years). Most participants were nurses, 58.7% (n=134), followed by other healthcare cadres, 33.7% (n=77), and doctors, 7.4% (n=17).

Fig. 2 shows the distribution of sleep times as recorded by Fitbit separated by age and sex. Entries of participants with sleep durations less than 3 hours (which included naps) were excluded. Across all participants n=434 with sleep data: mean number of entries: 99.83, median: 44.50. To enhance data quality and reliability, we restricted our analysis to participants with more than 40 valid sleep entries. This resulted in a final analytic sample of 228 participants, with a median entry count of 144.50 and a mean entry count of 179.26. Fig. 2 shows the distribution of sleep times, as recorded by Fitbit separated by age and sex. A pattern of shorter sleep durations for females was seen as they age above 23 years old. However, this pattern was less apparent in males, perhaps since they were 50% female participants than male participants.

Sleep onset, offset and midpoint times for the reported sleep episodes are shown in Fig. 3. It is interesting to note that there is a significant subpopulation of individuals who sleep during the day rather than the night. Because of the wide distributions of midsleep times between 10am and 8pm, we decided to test the hypothesis that this was due to different subpopulations. Separating out individuals based on their occupation, we see in Fig. 4 differences in the sleep onset, midsleep and sleep offset. We see a clear pattern whereby nurses more regularly work on night shifts and have a clear pattern and peak of midsleep on shifts of around 3pm. These effects were less pronounced for doctors, and a particular pattern of sleep during the day was largely absent from the rest of the workers.

We next conducted an in-depth analysis of wearable circadian metrics derived as in conducted with the social rhythm app. Although this app was not used by participants, we input the minute-by-minute sleep, step and heart rate data into the app code to determine previously validated circadian metrics^10–12^. As mentioned in the methods, this analysis focused on ~ 10% of the entire dataset since we could only fit 120,000,000 Fitbit derived metrics into memory from the unsorted MyDataHelps output. We also only included days where there were at least 120 heart rate entries, and for which either the heart rate circadian phase or central clock circadian phase was estimated to be within 2 hours. This later restriction is removed for comparison but still includes some data from 120 individuals for the heart rate phase and 145 individuals for the central clock phase. The resulting measurements yielded 619 days of heart rate measurements, 1568 days of central clock measurements and 1876 days where the sleep midpoint was measured with some data coming from most of the original participants.

Fig. 5a shows the distribution of the circadian phase as derived from the heart rate measurements following the algorithms of Kim et.al, Lee et.al^13,14^ and Bowman et.al^12^. The circular mean of the heart rate phases was 2.6 with a circular standard deviation of 6.1. This standard deviation was larger since a small population seemed to have a heart rate phase that was opposite to what was normally found. This measures the alerting circadian signals during the day. Fig. 5b shows the distribution of central clock phases, which are where a mathematical model that has been previously validated would predict the sleep midpoint would be, with respect to sleep midpoint^13,14^. This shows the phase of the circadian signal which promotes sleep. The circular mean of this measurement was 5.3 with a standard deviation of 2.8. Fig. 5c shows the sleep midpoints which were included in our analysis which had a circular mean of 2.95 and a standard deviation of 2.2. This corresponded primarily with individuals who had a midsleep during the day particularly since circadian signals from night shift workers tend to have large, estimated errors and thus were excluded as mentioned previously.

Fig. 5d and 5e show the relationship between sleep midpoint and the two circadian metrics. While these are consistent with previous studies, two trends emerged. First, the heart rate circadian phase typically preceded the sleep midpoint. While this is earlier than found in US medical interns,^6^ this population is older and the difference is consistent with the earlier circadian phase seen when age is considered^11^. Additionally, the central clock circadian phase with respect to sleep midpoint was typically later (by approximately ~2 hours) than seen in the US interns.

We plot the histogram of the differences between sleep midpoint and the two circadian metrics, as we see the difference between the circadian metrics (internal misalignment) in Fig. 5f. We can see the small population with misaligned (8 to 12 hour difference) heart rate phase with respect to sleep midpoint. A similar bump in the internal misalignment was probably not seen since it might induce a large uncertainty in the central clock phase^13^.

Finally, we found 10 examples in the data where there was enough sequential data to plot actograms, which are common in the circadian field. These plots show counts of steps (black, interpolated if data is missing) over time. Subsequent line represents subsequent days, and the data is double plotted (48 hours of data shown on each line) to show patterns. The heart rate phase (red) and central clock phase (blue) are shown. These individuals show circadian patterns that are consistent with what is seen in other populations, however, as discussed earlier, in this population the heart rate circadian phase typically precedes the central clock phase. We additionally note that Fig. 6 subject h) had the clearest misalignment between sleep midpoint and central clock phase.

This study provides the first longitudinal, wearable-based characteristics of sleep and circadian rhythms among HCWs in Kenya, demonstrating the feasibility of continuous digital sleep monitoring in a resource-limited setting. The ability to capture reliable, objective sleep data over extended periods highlights the potential of wearable technologies to generate scalable, real-world evidence in LMICs, where such data remain scarce.

Overall, sleep patterns in this cohort were comparable to those reported in high-income settings^15–17^ particularly in terms of total sleep duration; however, a substantial burden of insufficient and irregular sleep persists, especially among shift-working HCWs. In contrast, non-shift workers exhibited more regular sleep patterns, reinforcing the central role of occupational demands in shaping sleep health. Notably, a subset of participants demonstrated internal circadian desynchrony, prospectively driven by delayed circadian phase, supporting circadian misalignment as a key pathway through shift work. Importantly, this study illustrates the added value of wearable-derived circadian metrics in detecting physiological disruptions that are not captured by traditional self-report methods. These findings position digital biomarkers as promising tools for early identification of sleep and circadian dysfunction in high-risk occupational groups.

Despite limitations including a demographically skewed towards the urban HCWs setting, absence of subjective sleep measures, the results provide critical baseline evidence for occupational sleep health in LMICs. Future research should include more diverse populations, leverage actogram analysis to enhance monitoring, diagnosis, and treatment of sleep and circadian rhythm disorders, integrate both subjective and objective assessment measures, and strengthen local data infrastructure to support sustainable digital health research. Nonetheless, these findings underscore the practicality and translational potential of wearable sleep monitoring in LMICs and highlight an urgent need for context-specific, scalable interventions to improve sleep health and workforce well-being.

## Methods

### Ethics statement

The Aga Khan University, Institutional Scientific and Ethics Review Committee and Kenyatta University Teaching Referral and Research Committee approved the study (2021/IERC-166(V2) regulatory approvals from the National Commission for Science Technology and Innovation (NACOSTI/P/23/23050), Nairobi County and Nairobi Metropolitan Services (EOP/NMS/HS/167). The study was conducted in accordance with the Declaration of Helsinki and electronic informed consent was obtained from all the participants.

### Participants

**Fig. 1:**
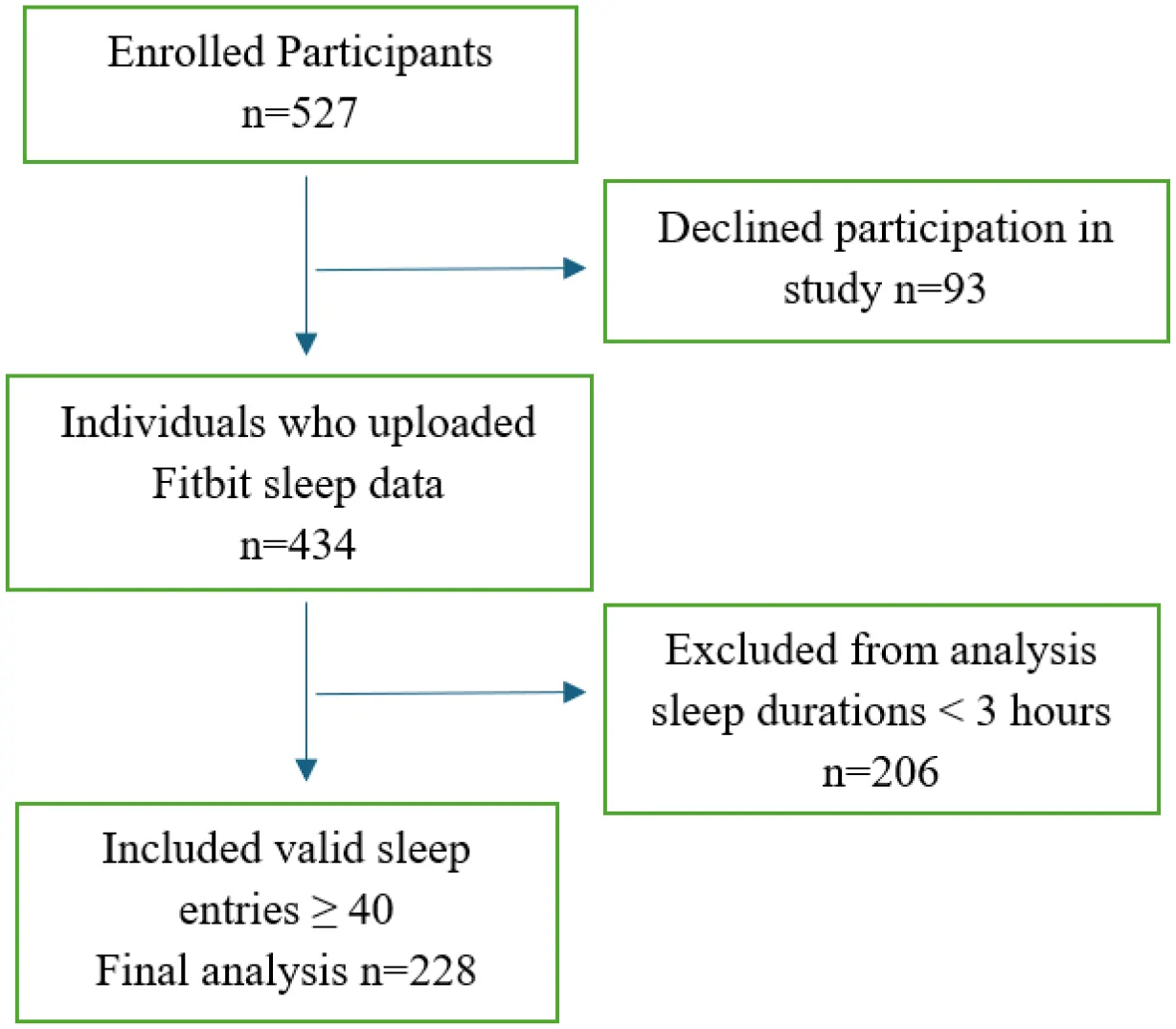
Study flow diagram detailing healthcare workers from enrollment to analysis

A total of 527 participants were enrolled from five healthcare facilities (one private and four public), of whom 434 uploaded sleep data (Fig. 1). During the 12-month period (April 2023 - November 2024), we tracked the number of valid sleep entries defined as nights when participants wore the Fitbit Inspire 2^™^ during sleep and successfully transmitted data.

### Materials

The mobile application, MyDataHelps^™^ (Care Evolution, Ann Arbor, Michigan, USA), was used to collect and integrate active (captured information on age, gender, marital status, cadre, place of residence, and level of education and quarterly surveys) and passive (wearable) data. The app was available on Android and iOS operating systems.

### Wearable device

**Fig. 5:**
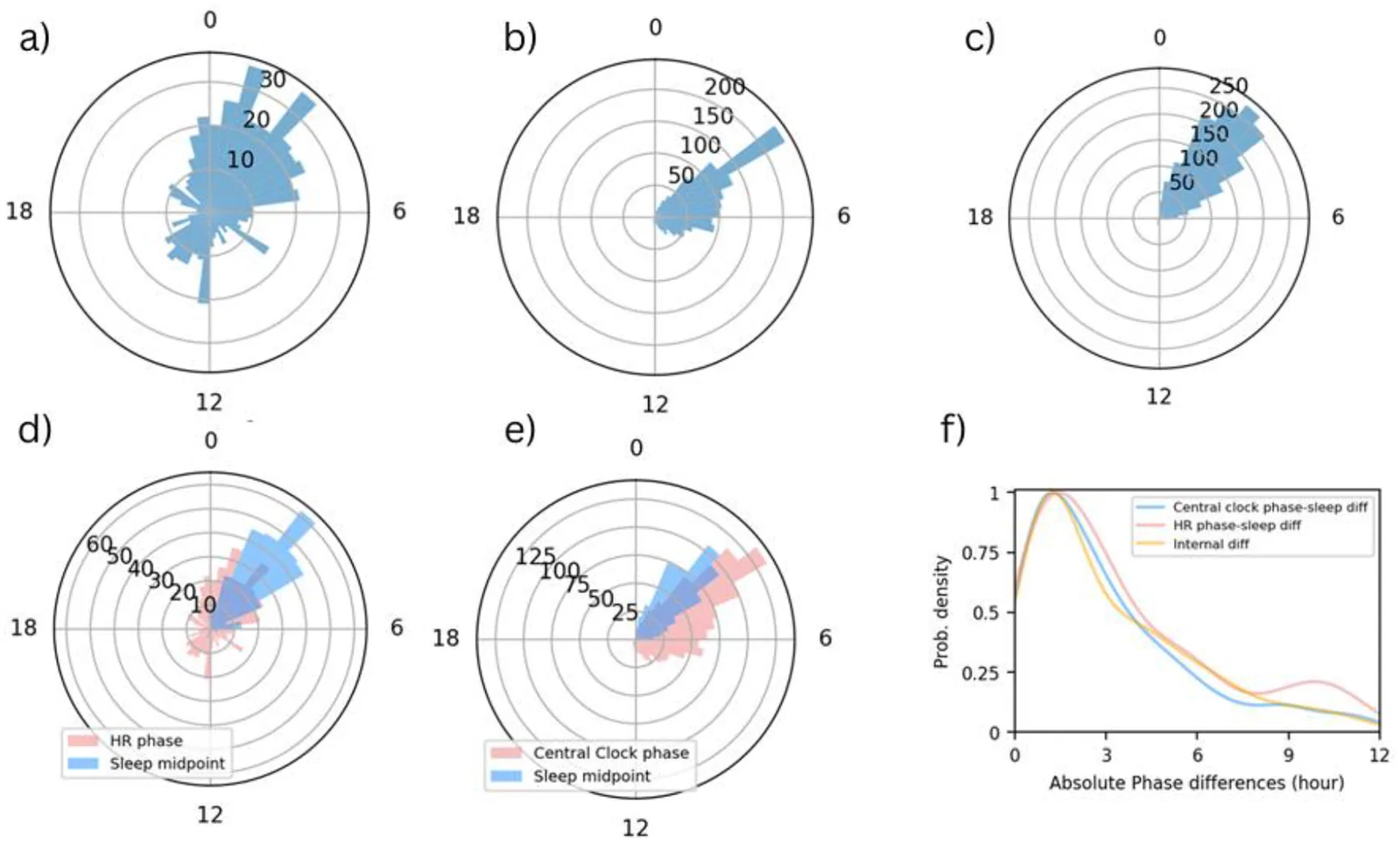
Circadian metrics a) Circular histogram of the phases of the circadian rhythm in heart rate (CRHR). b) Circular histogram of the phases of the central circadian clock (CRCO). c) Circular histogram of the sleep midpoints. Metrics were similar to US cohorts except for a delayed circadian phase in the CRCO. Only data where the CRCO or CRHR were estimated within +− 2 hours were included. d) Comparison between the CRHR and midsleep. e) Comparison between the CRCO and midsleep. f) Histograms of these differences between these metrics. These differences were similar, indicating that there were no systemic differences, however, a smaller discrepancy was seen in CRHR-midsleep due to some out of phase heart rate rhythms.

**Fig. 6:**
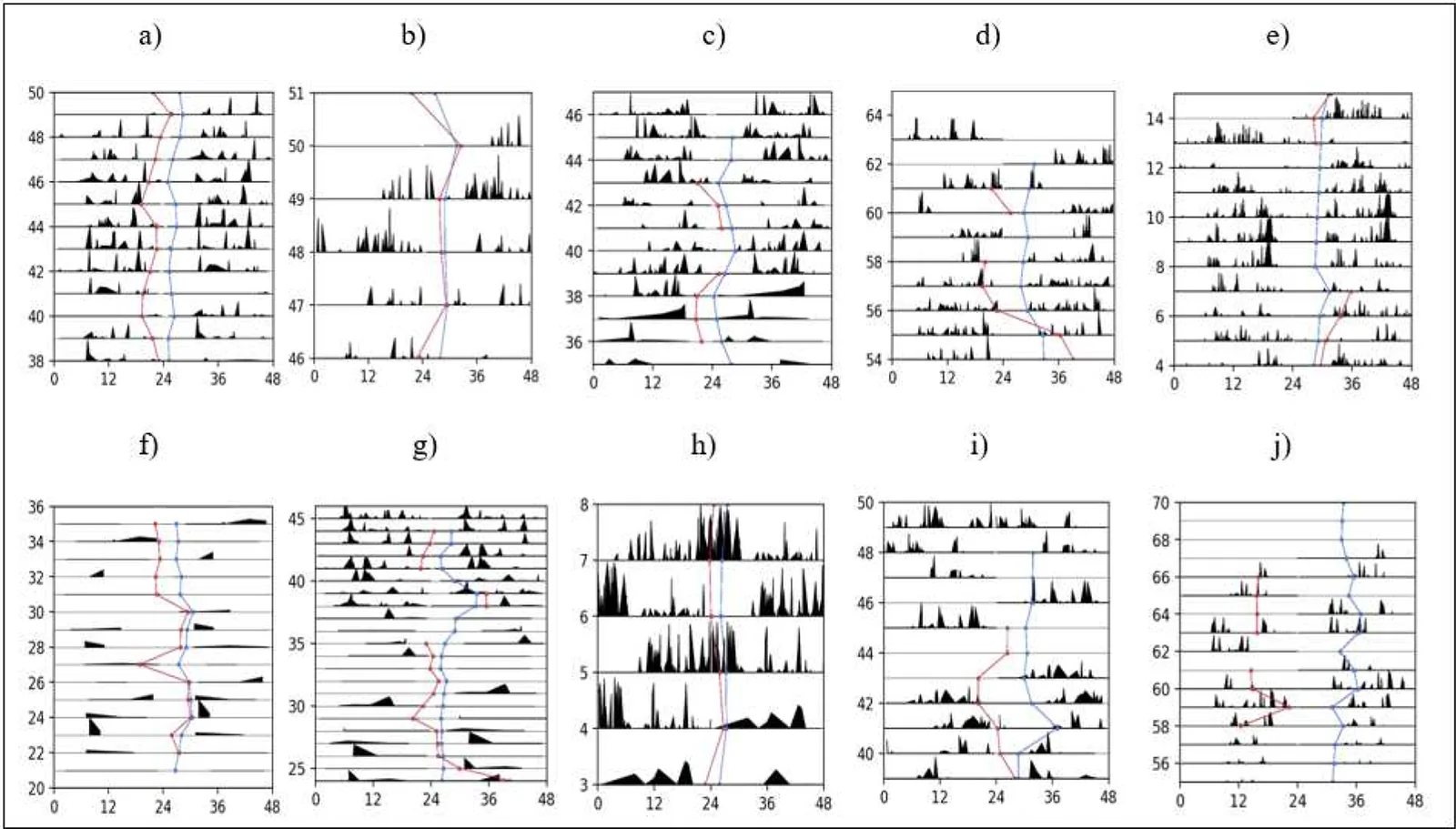
Actograms (a-j) illustrating individual sleep–wake activity patterns among healthcare workers, with heart rate displayed in red and central circadian phase shifts represented in blue illustrating temporal changes including phase shifts of up to 24 hours. Y-axis represents number of days; X-axis indicates time in hours.

Participants wore a Fitbit Inspire 2^™^ (Fitbit Inc, San Francisco, CA, USA), a wearable fitness tracker wristband, to continuously track daily activity levels and sleep. The device automatically connects via Bluetooth and transfers data to the MyDataHelps mobile application via the Fitbit App. The Fitbit Inspire 2^™^ enabled tracking of sleep stages, including time spent awake, in light sleep, deep sleep, and rapid eye movement (REM) sleep, as well as distinguishing between sleep and wake states and heart rate variability. Specifically, activity levels and heart rate (Fig 6) were measured via the device’s built-in accelerometer and photoplethysmography sensor. Examples of changes in activity levels over time are shown as actograms in Fig 6. Due to challenges associated with handling the substantial volume of data in the database, we only sampled the top 120,000,000 entries of the intraday data utilizing the SQL top command. However, our analysis focused on step, heart rate and sleep derived circadian metrics, so many of these data points were not needed. More entries could not be processed since the data from MyDataHelps were unsorted and this was the maximum amount of data that could be fit into computer memory. This approach yielded extensive data for many individuals, and when there was at least an amount of continuous heart rate data, as described in Bowman et.al^12^, we estimated circadian phase from heart rate CRHR. For all individuals we estimated the central circadian phase CRCO via the algorithms described in Huang^11^ sleep, based on the timings of sleep. The difference between these two circadian measures and their difference from the midpoint of sleep is shown in Fig 5.

### Procedure

After completing the consent process and initial survey through the *MyDataHelps* app, participants received a Fitbit Inspire 2^™^ device. They were asked to complete follow-up online surveys at months 3, 6, 9, and 12. Participants were instructed to wear the device continuously, synchronize it with their smartphones, and received quarterly compensation (Kenya Shilling 500 equivalent US $3.83) to cover internet bundles costs. Detailed study design and protocol information can be found in a previous publication^18,19^.

### Statistical analysis

The statistical analyses and data processing were conducted in R (version 4.5.1) and presented as means – SD and frequencies and percentages. Descriptive statistics were used to analyze and describe respondents’ characteristics. To ensure the accuracy and reliability of the dataset, a rigorous data pre-processing protocol was implemented. Incomplete or erroneous recordings, as well as implausible values (sleep durations <3 hours) were excluded. Non-wear periods were identified through device-based detection algorithms and removed from the dataset. All time-stamped data were standardized to a common time zone to facilitate accurate circadian comparisons. Quality control procedures included filtering out data from malfunctioning devices and sessions with synchronization errors. These steps minimized noise measurement, reduced potential sources of bias, and ensured that the resulting dataset accurately reflected participants’ habitual sleep patterns, thereby strengthening the internal validity and reproducibility of the study findings. In addition to the homeostatic effects of sleep where the waking day increases sleep pressure that is relieved during sleep, circadian rhythms play a prominent role in our understanding of sleep^20^. Beyond sleep, we considered two circadian metrics, an estimate of the circadian phase based on mathematical models, activity and sleep data, circadian rhythm in the central oscillator (CRCO) we directly measured circadian rhythm in heart rate (CRHR). Comparing sleep midpoint with the two metrics CRCO and CRHR provides an estimate of whether sleep timing is occurring at the proper phases.

## Figures and Tables

**Fig. 2:**
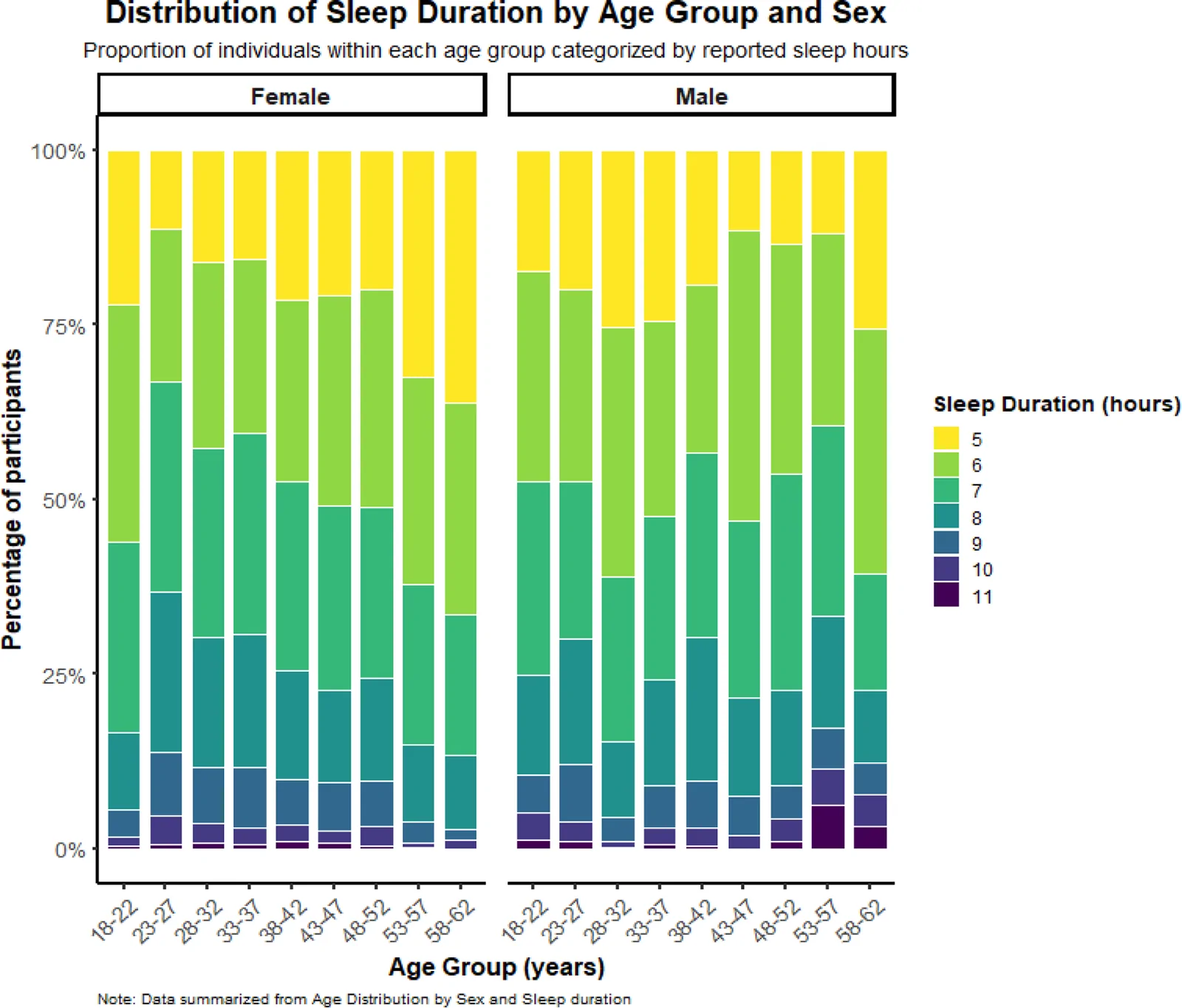
Sleep duration by age group and sex (n=221). Total sleep times of five hours or more as reported by Fitbit.

**Fig. 3:**
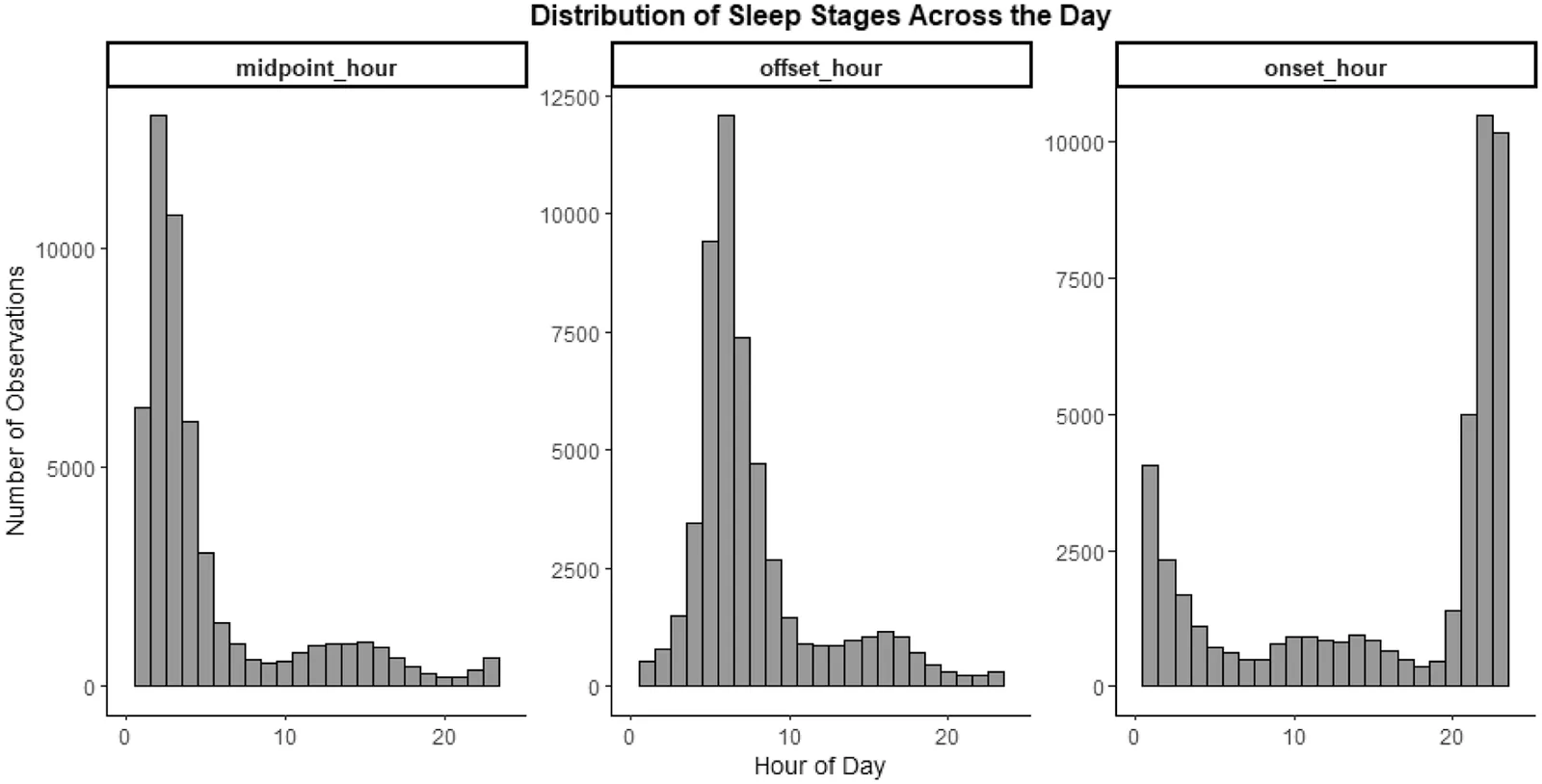
Distribution of sleep stages

**Fig.4:**
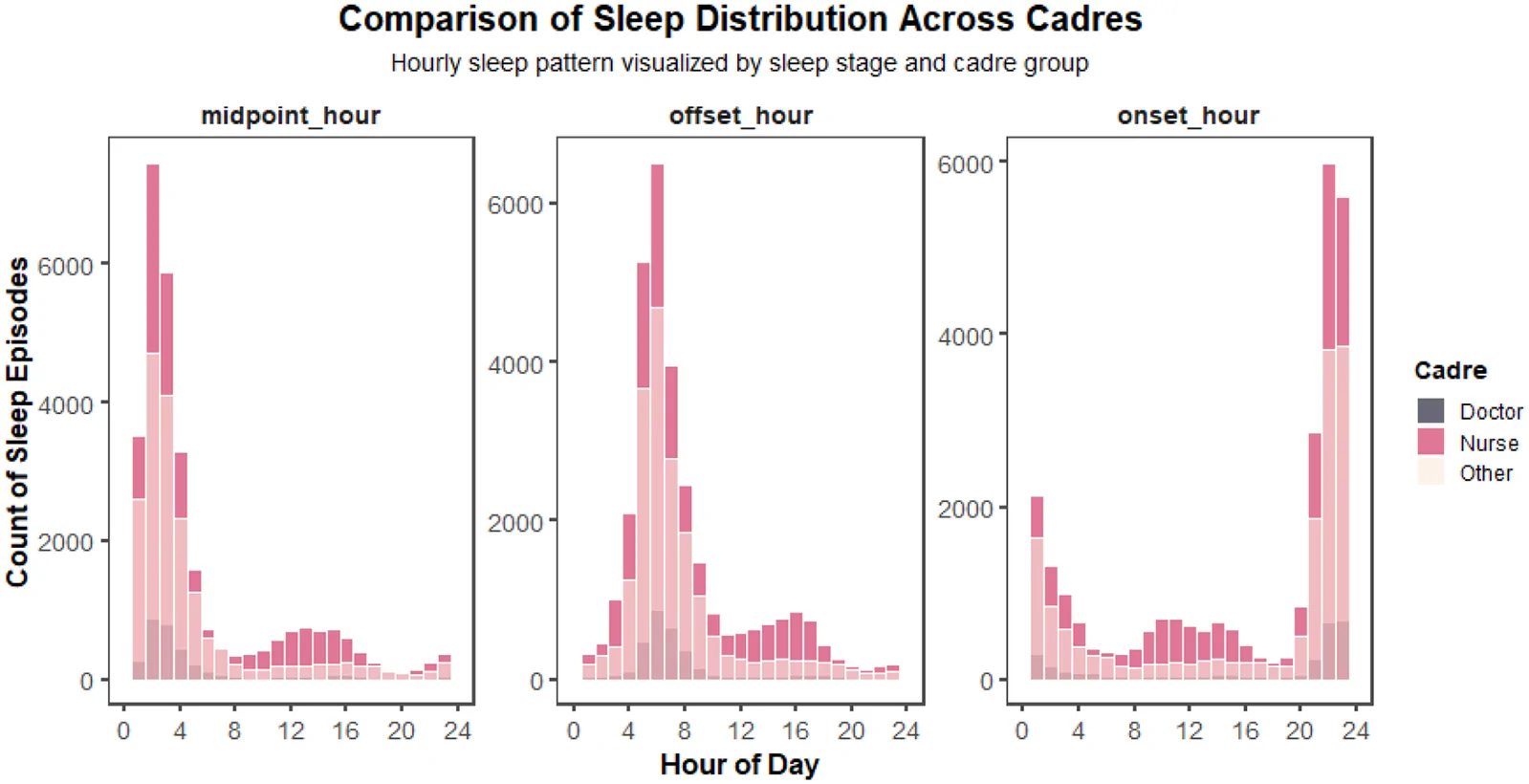
Sleep distribution per cadre (doctors n=17, nurses n=137, others n=77)

## Data Availability

The de-identified data from this study that supports our findings are available from the authors upon reasonable request.

## Code Availability

A MATLAB version of the code for circadian analysis can be found here^6,12^. The Social Rhythms app is available for download on Google Play or Apple App stores.
